# Supplementary material for: Synthesis of Novel Spiro-Tetrahydroquinoline Derivatives and Evaluation of Their Pharmacological Effects on Wound Healing
Source: Int J Mol Sci. 2021 Jun 10;22(12):6251. doi: 10.3390/ijms22126251 (PMC8230376; doi:10.3390/ijms22126251)
Supplement: Supplementary file 1 [file ijms-22-06251-s001.zip › ijms-1230978-supplementary.pdf]

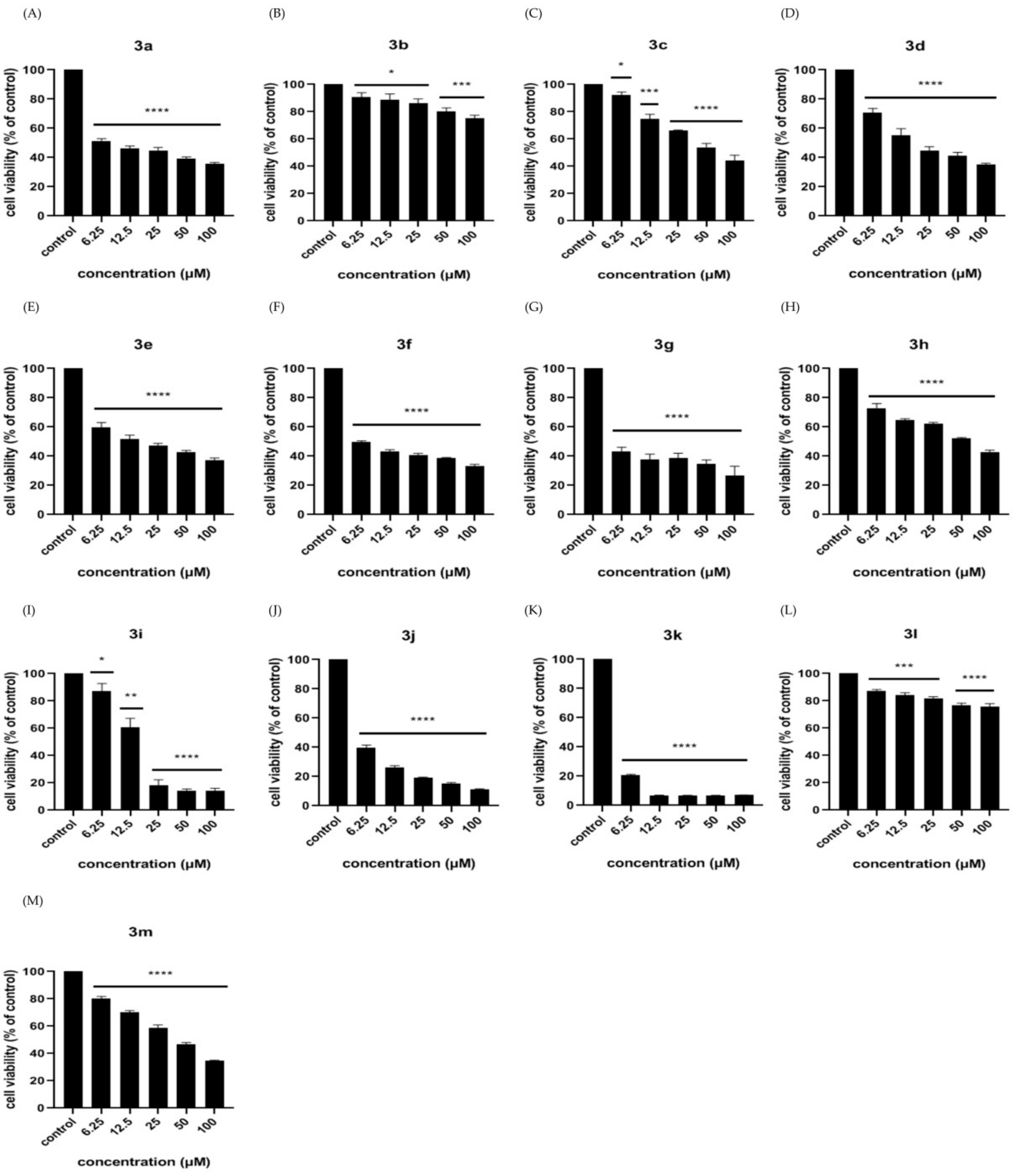


**Figure S1.** Effects of (A) **3a,** (B) **3b,** (C) **3c,** (D) **3d,** (E) **3e,** (F) **3f,** (G) **3g,** (H) **3h,** (I) **3i,** (J) **3j,** (K) **3k,** (L) **3l and** (M) **3m** on cell viability in HaCaT cell. The cells were treated with different concentrations of compounds for 24 h. The cell viabilities were measured by MTT assay as Table 3. Values are expressed as mean ± SD for triplicate samples and expressed as a percentage of control (set as 100 %). * $p < 0.05$, ** $p < 0.01$, *** $p < 0.001$, **** $p < 0.0001$.

(A)

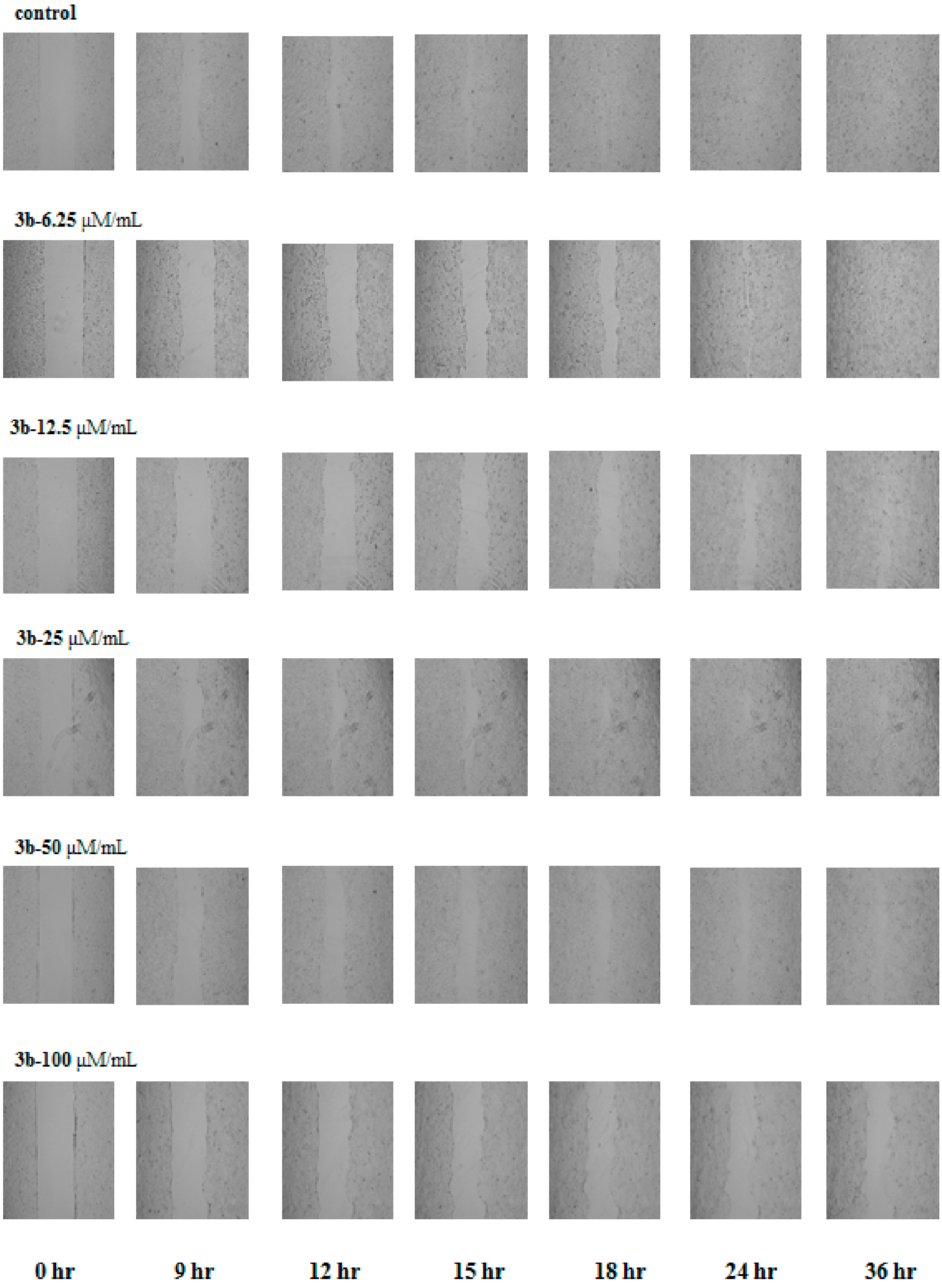
control
3b-6.25 µM/mL
3b-12.5 µM/mL
3b-25 µM/mL
3b-50 µM/mL
3b-100 µM/mL
0 hr
9 hr
12 hr
15 hr
18 hr
24 hr
36 hr

(B)

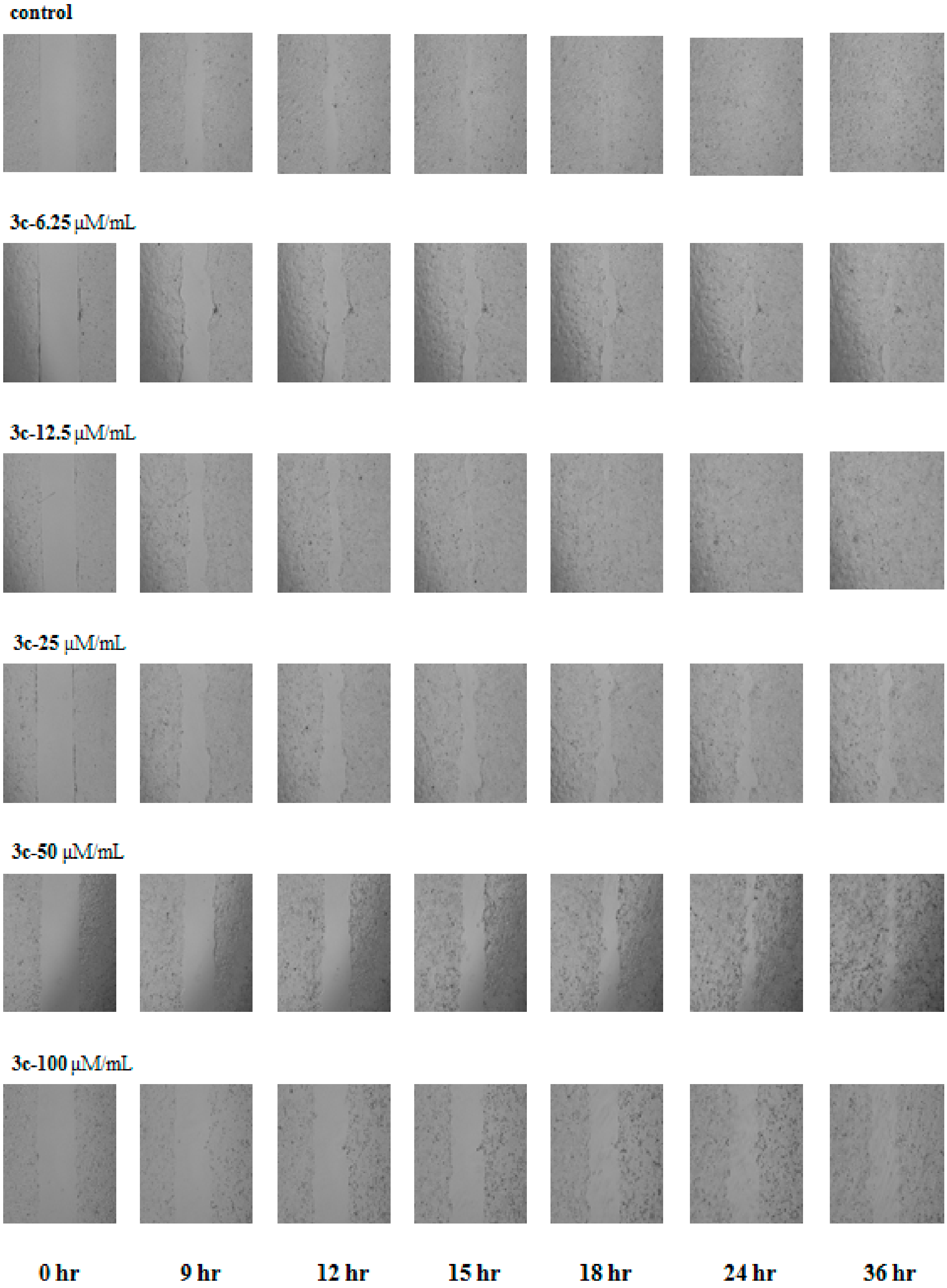
control
3c-6.25 μM/mL
3c-12.5 μM/mL
3c-25 μM/mL
3c-50 μM/mL
3c-100 μM/mL
0 hr
9 hr
12 hr
15 hr
18 hr
24 hr
36 hr

(C)

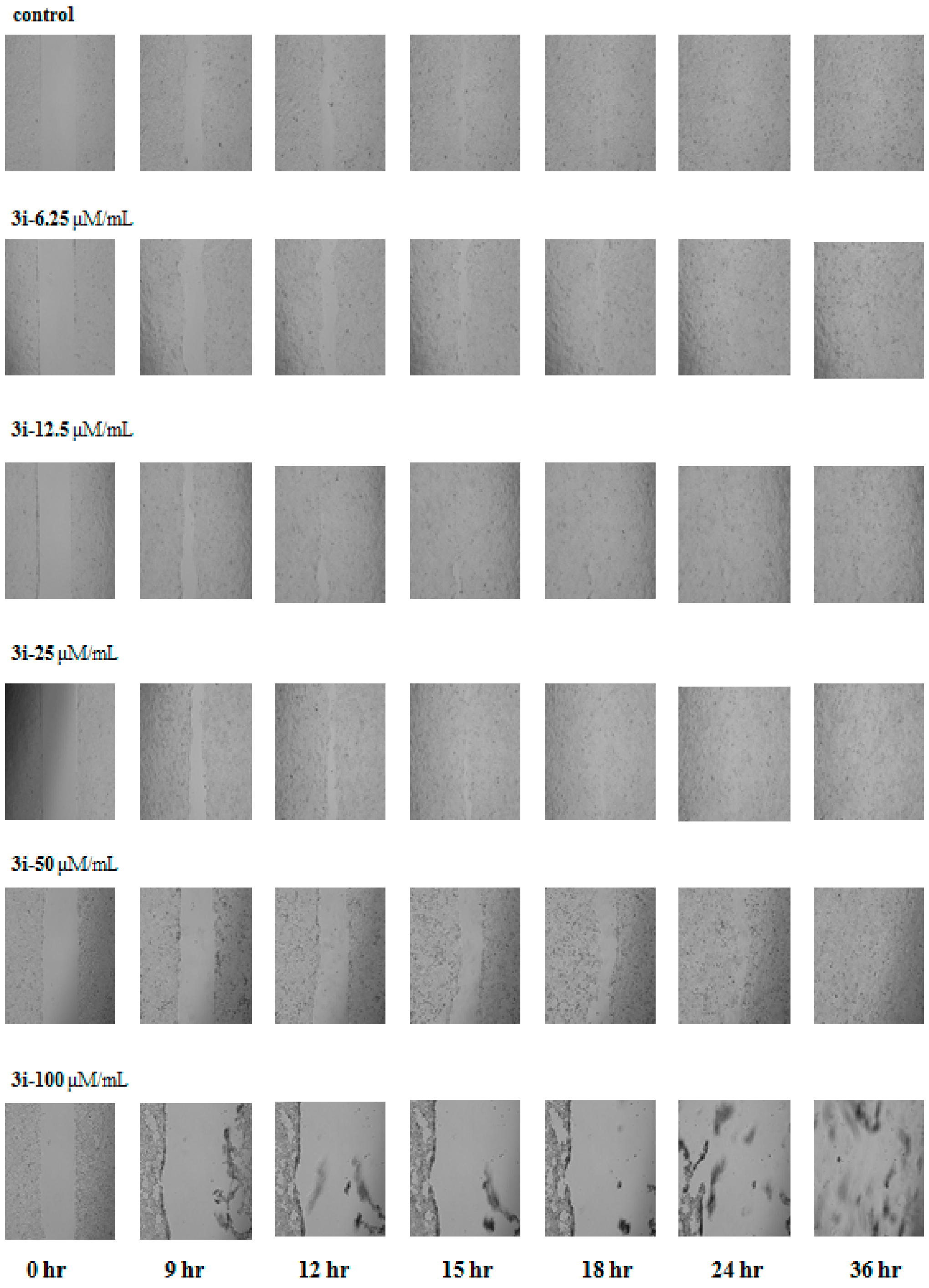
control
3i-6.25 µM/mL
3i-12.5 µM/mL
3i-25 µM/mL
3i-50 µM/mL
3i-100 µM/mL
0 hr
9 hr
12 hr
15 hr
18 hr
24 hr
36 hr

(D)

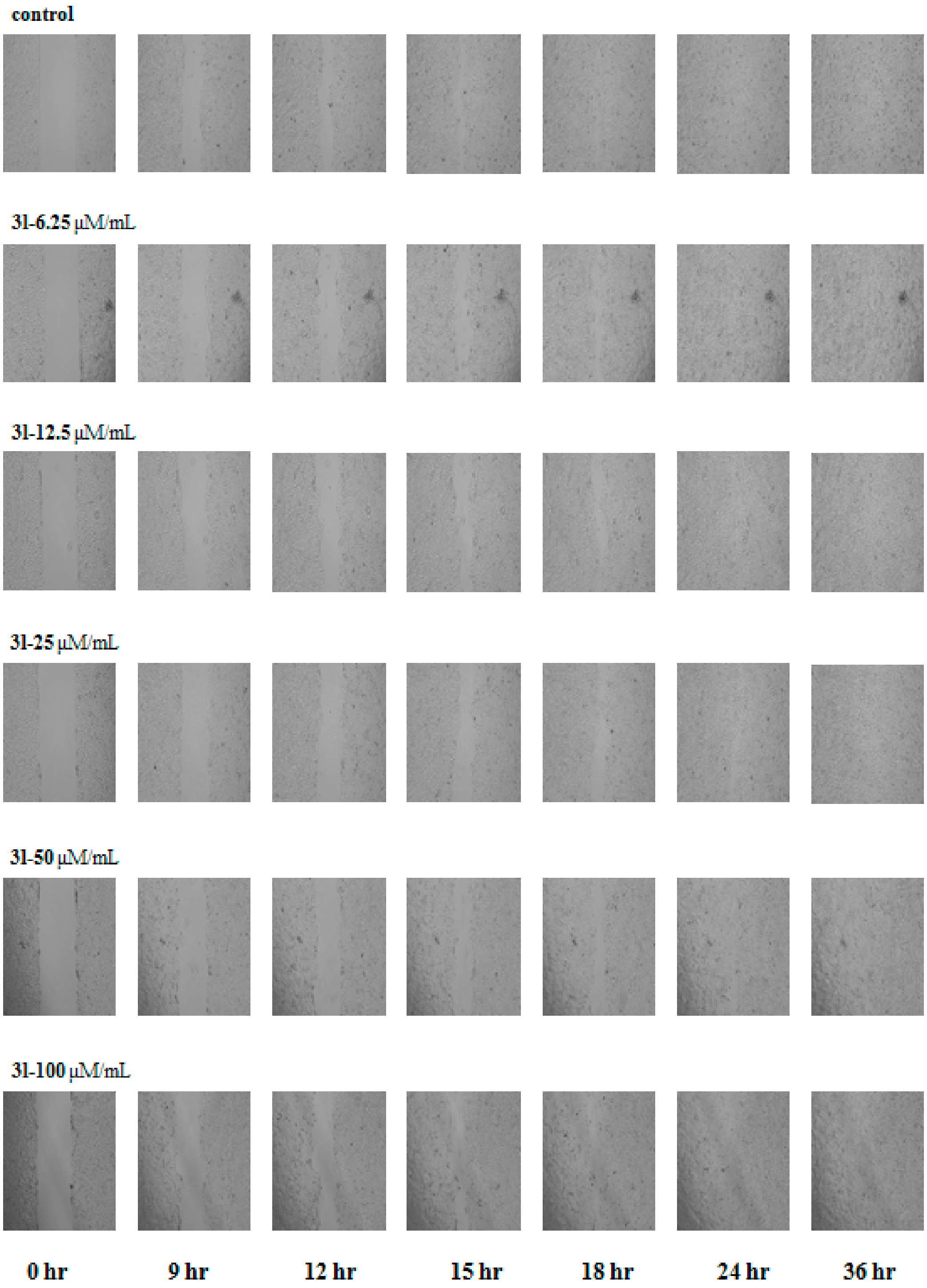
control
3l-6.25 μM/mL
3l-12.5 μM/mL
3l-25 μM/mL
3l-50 μM/mL
3l-100 μM/mL
0 hr
9 hr
12 hr
15 hr
18 hr
24 hr
36 hr

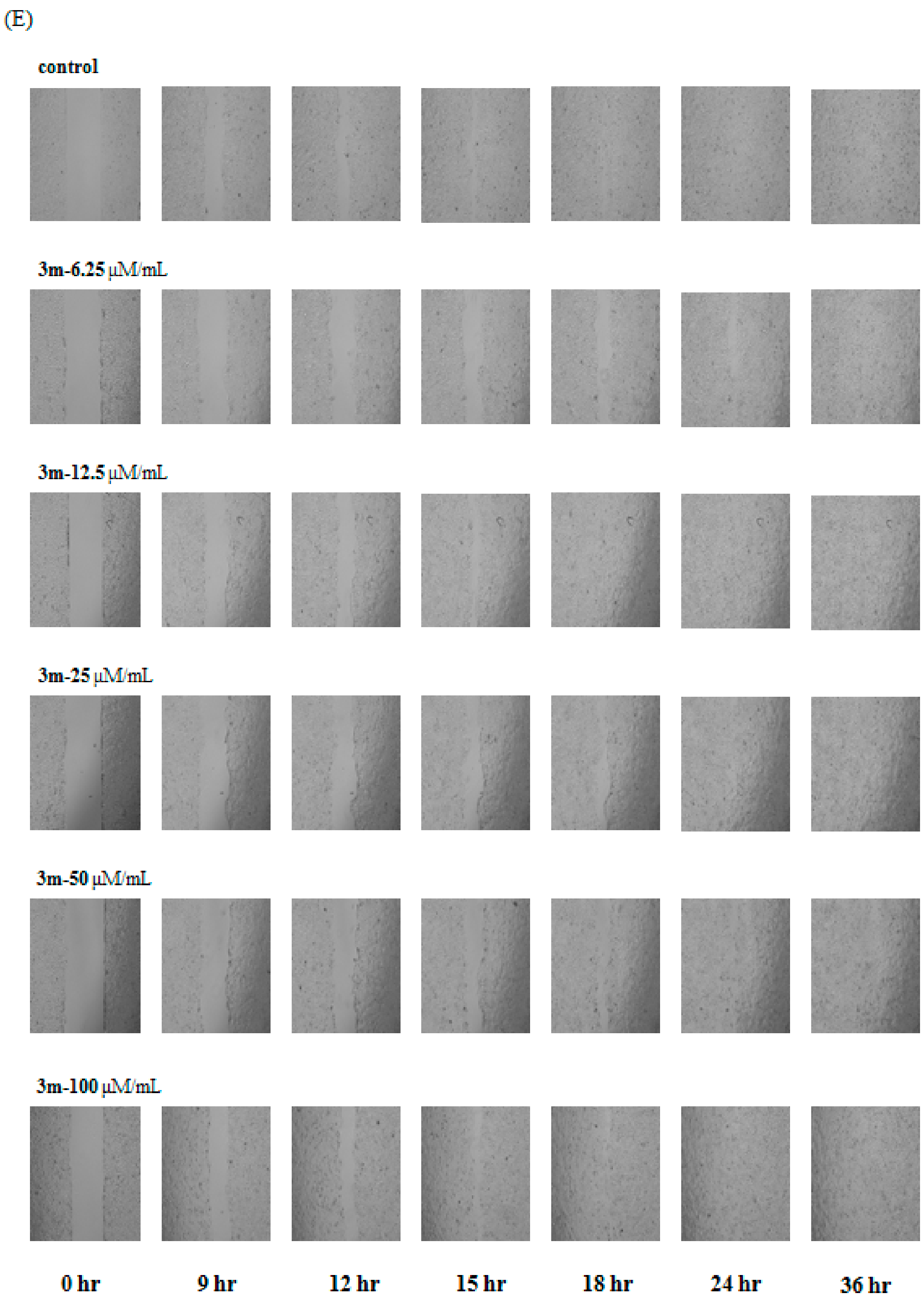


**Figure S2.** Scratch analysis rate of HaCaT cells. (**A**) Cell migration photos of **3b** (**B**) Cell migration photosof **3c**, (**C**) Cell migration photos of **3i**, (**D**) Cell migration photos of **3l**, (**E**) Cell migration photos of **3m**.

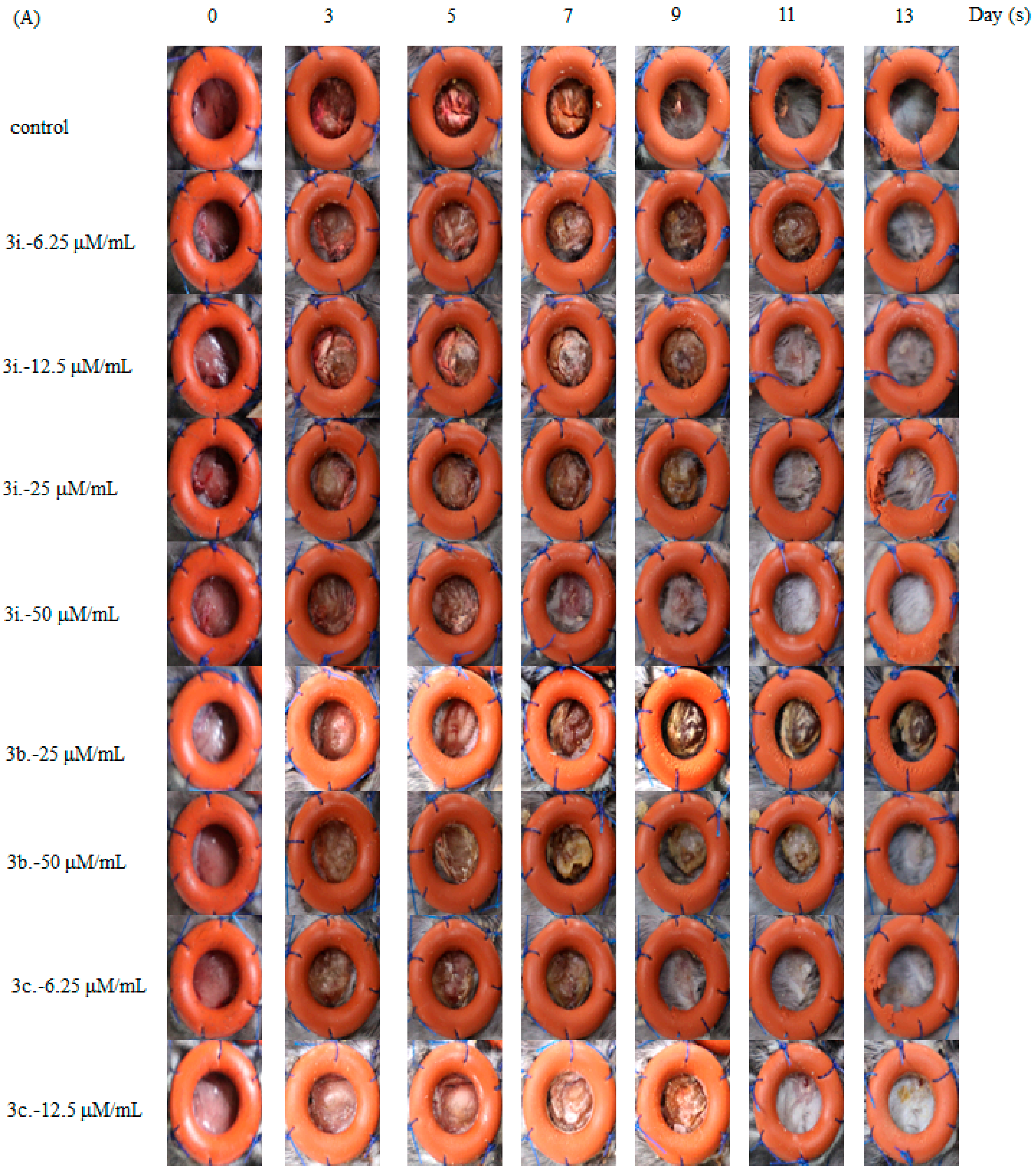


**Figure S3.** C57BL/6 mouse simulated wound trauma animal model. Photographs of mouse wounds were observed at 0, 3, 5, 7, 9, 11 and 13 days.

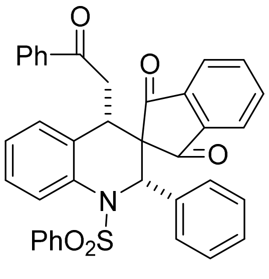


3a

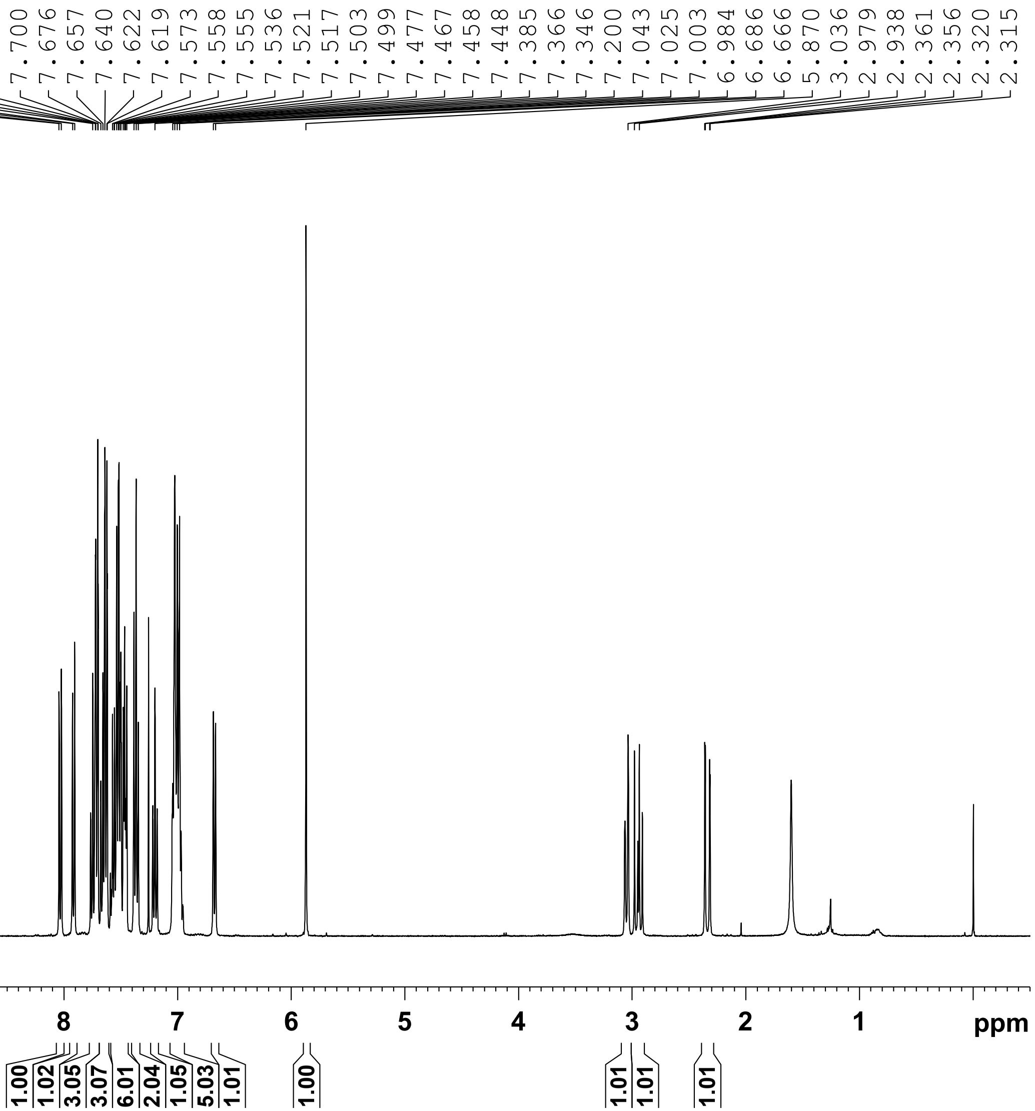


```
Current Data Parameters
NAME          no substitu
EXPNO                   1
PROCNO                  1

F2 - Acquisition Parameters
Date_            20180811
Time                14.40
INSTRUM             spect
PROBHD   5 mm BBO BB-1H
PULPROG              zg30
TD                  32768
SOLVENT             CDCl3
NS                     16
DS                      0
SWH              7246.377 Hz
FIDRES           0.221142 Hz
AQ              2.2609921 sec
RG                    114
DW                 69.000 usec
DE                   6.50 usec
TE                  296.3 K
D1             2.00000000 sec
TD0                     1

======== CHANNEL f1 ========
NUC1                   1H
P1                  15.00 usec
PL1                  2.20 dB
SFO1         400.1324008 MHz

F2 - Processing parameters
SI                  16384
SF           400.1300110 MHz
WDW                    EM
SSB      0
LB       0 Hz
GB       0
PC                   1.00
```

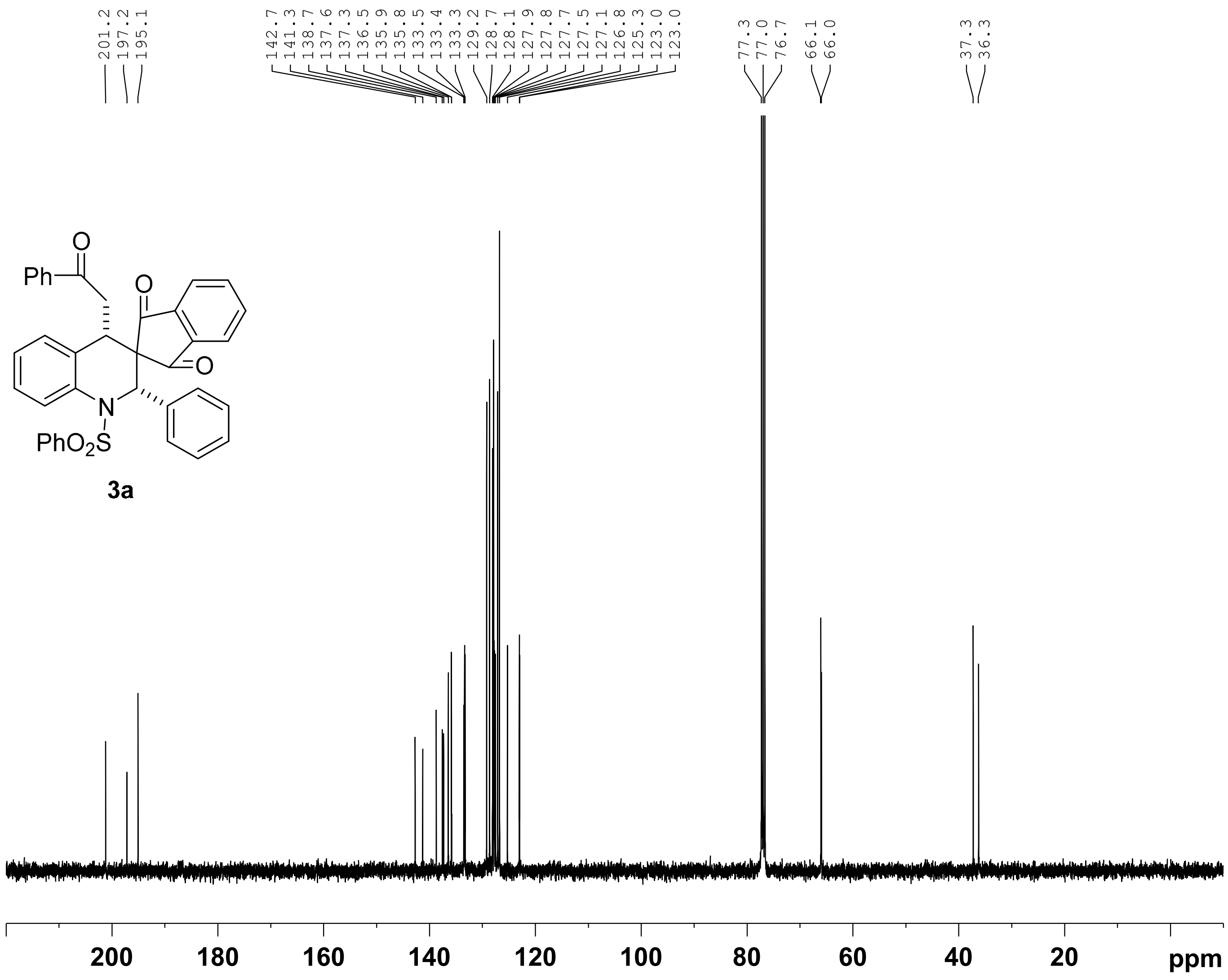


```
Current Data Parameters
NAME          no substitu
EXPNO                  13
PROCNO                  1

F2 - Acquisition Parameters
Date_            20180811
Time                14.42
INSTRUM             spect
PROBHD   5 mm BBO BB-1H
PULPROG           zgpg30
TD                 32768
SOLVENT            CDCl3
NS                   763
DS                     0
SWH           24038.461 Hz
FIDRES         0.733596 Hz
AQ            0.6815744 sec
RG                  1024
DW                20.800 usec
DE                  6.50 usec
TE                 296.2 K
D1            2.00000000 sec
D11           0.03000000 sec
TD0                    1

======== CHANNEL f1 ========
NUC1                 13C
P1                 10.00 usec
PL1                 7.50 dB
SFO1         100.6233325 MHz

======== CHANNEL f2 ========
CPDPRG[2         waltz16
NUC2                  1H
PCPD2              90.00 usec
PL2                 1.40 dB
PL12               17.50 dB
PL13               20.50 dB
SFO2         400.1316005 MHz

F2 - Processing parameters
SI                 32768
SF           100.6127741 MHz
WDW                   EM
SSB      0
LB                  1.00 Hz
GB       0
PC                  1.00
```

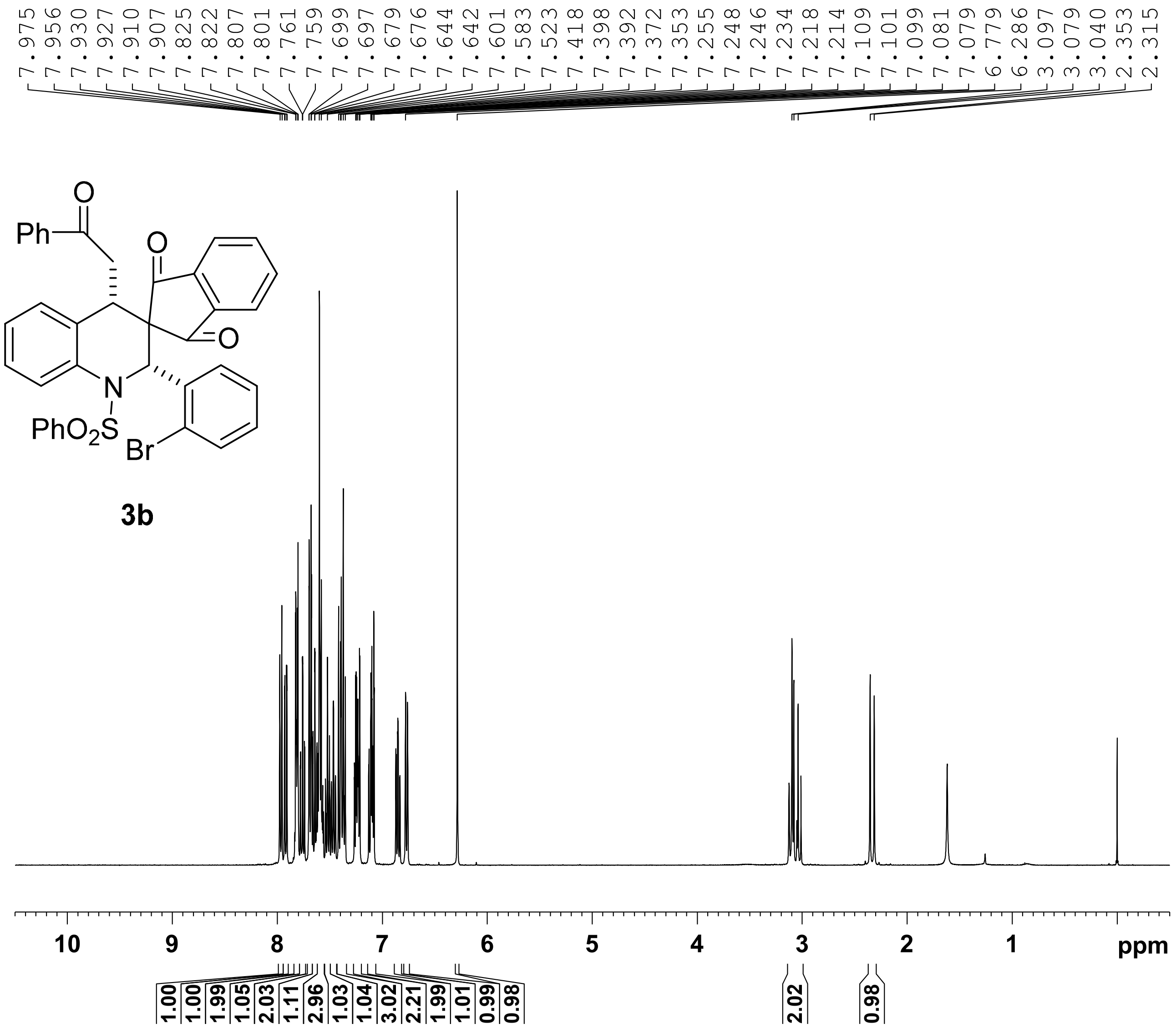


**3b**

```
Current Data Parameters
NAME                 2-Br
EXPNO                   1
PROCNO                  1

F2 - Acquisition Parameters
Date_            20180731
Time                 7.32
INSTRUM             spect
PROBHD   5 mm BBO BB-1H
PULPROG              zg30
TD                  32768
SOLVENT             CDCl3
NS                     16
DS                      0
SWH              7246.377 Hz
FIDRES           0.221142 Hz
AQ              2.2609921 sec
RG                    114
DW                 69.000 usec
DE                   6.50 usec
TE                  297.2 K
D1             2.00000000 sec
TD0                     1

======== CHANNEL f1 ========
NUC1                   1H
P1                  15.00 usec
PL1                  2.20 dB
SFO1          400.1324008 MHz

F2 - Processing parameters
SI                  16384
SF            400.1300115 MHz
WDW                    EM
SSB       0
LB        0 Hz
GB        0
PC                   1.00
```

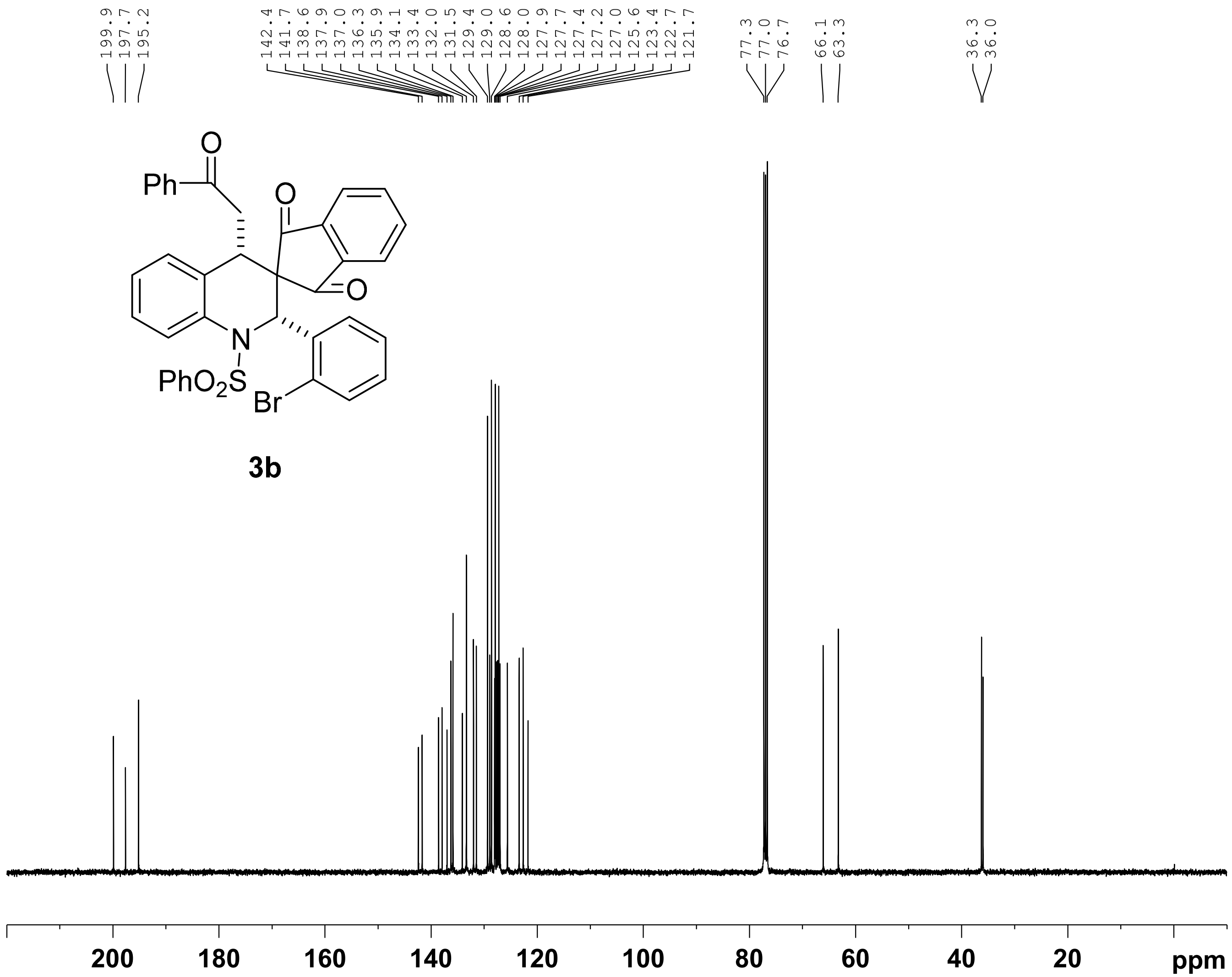


```
Current Data Parameters
NAME                2-Br
EXPNO                 13
PROCNO                 1

F2 - Acquisition Parameters
Date_           20180731
Time                7.34
INSTRUM            spect
PROBHD   5 mm BBO BB-1H
PULPROG          zgpg30
TD                 32768
SOLVENT            CDCl3
NS                  2491
DS                     0
SWH            24038.461 Hz
FIDRES          0.733596 Hz
AQ             0.6815744 sec
RG                  2048
DW                20.800 usec
DE                  6.50 usec
TE                 297.2 K
D1            2.00000000 sec
D11           0.03000000 sec
TD0                    1

======== CHANNEL f1 ========
NUC1                 13C
P1                 10.00 usec
PL1                 7.50 dB
SFO1         100.6233325 MHz

======== CHANNEL f2 ========
CPDPRG[2         waltz16
NUC2                  1H
PCPD2              90.00 usec
PL2                 1.40 dB
PL12               17.50 dB
PL13               20.50 dB
SFO2         400.1316005 MHz

F2 - Processing parameters
SI                 32768
SF           100.6127759 MHz
WDW                   EM
SSB      0
LB                  1.00 Hz
GB       0
PC                  1.00
```

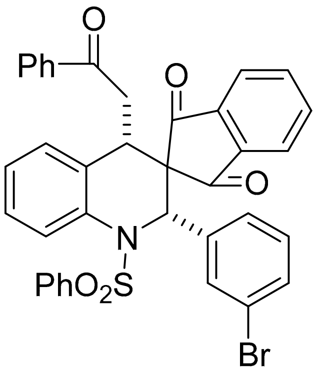

**3c**

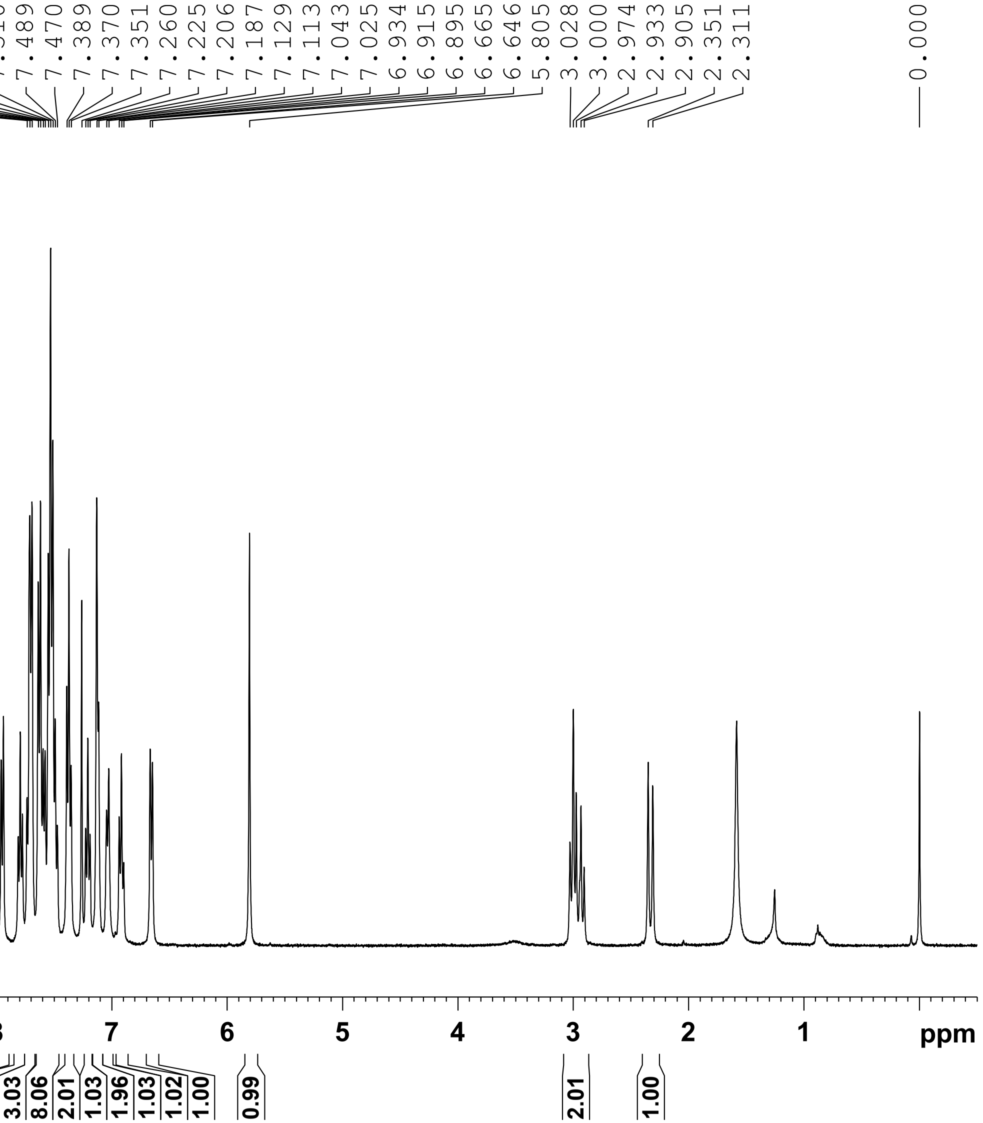


```
Current Data Parameters
NAME                 3-Br
EXPNO                   1
PROCNO                  1

F2 - Acquisition Parameters
Date_            20180731
Time_               21.23
INSTRUM             spect
PROBHD   5 mm BBO BB-1H
PULPROG              zg30
TD                  32768
SOLVENT             CDCl3
NS                     16
DS                      0
SWH              7246.377 Hz
FIDRES           0.221142 Hz
AQ              2.2609921 sec
RG                    114
DW                 69.000 usec
DE                   6.50 usec
TE                  296.8 K
D1             2.00000000 sec
TD0                     1

======== CHANNEL f1 ========
NUC1                   1H
P1                  15.00 usec
PL1                  2.20 dB
SFO1          400.1324008 MHz

F2 - Processing parameters
SI                  16384
SF            400.1300098 MHz
WDW                    EM
SSB      0
LB       0 Hz
GB       0
PC                   1.00
```

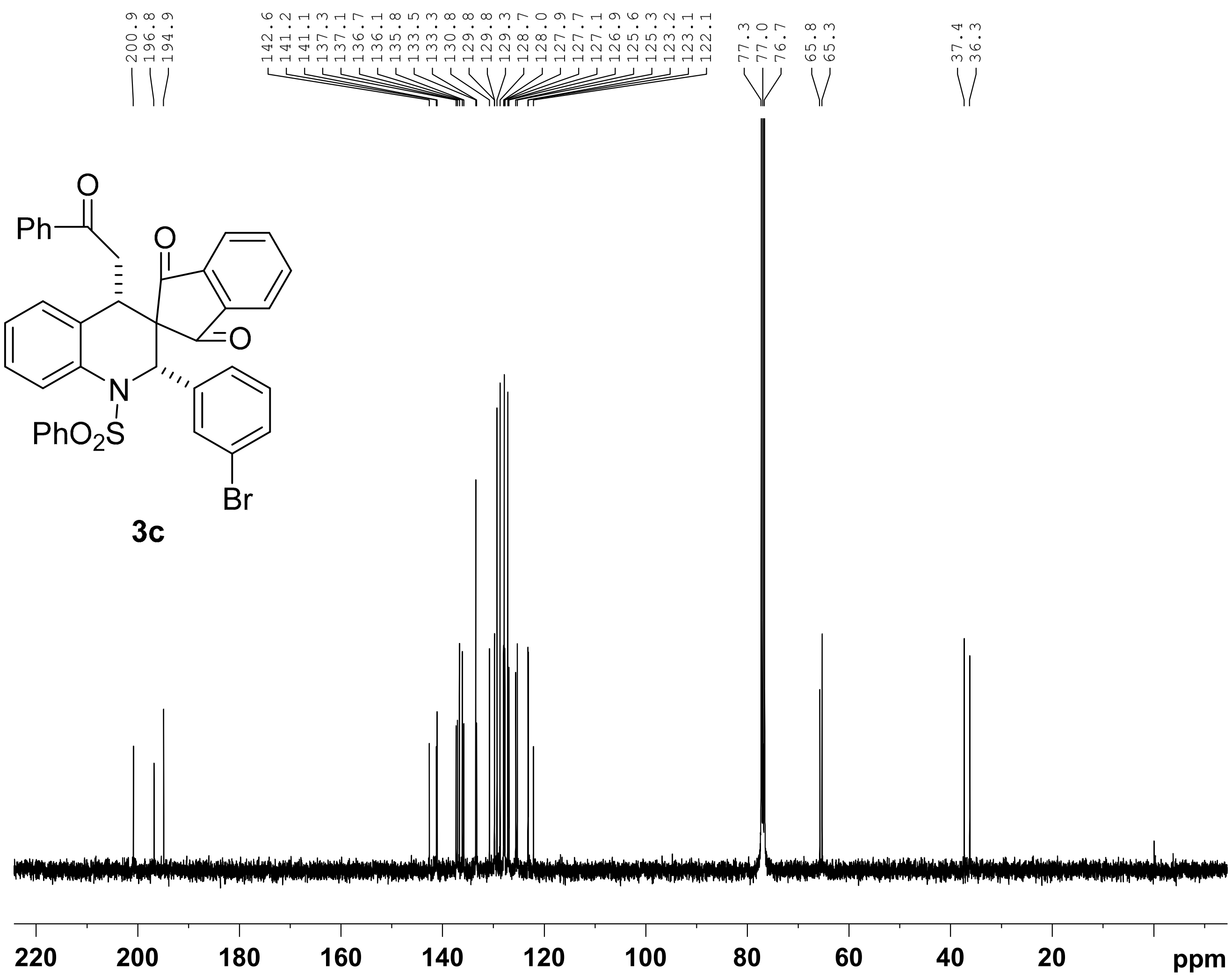


```
Current Data Parameters
NAME                 3-Br
EXPNO                  13
PROCNO                  1

F2 - Acquisition Parameters
Date_            20180801
Time                 8.08
INSTRUM             spect
PROBHD   5 mm BBO BB-1H
PULPROG           zgpg30
TD                  32768
SOLVENT             CDCl3
NS                   2856
DS                      0
SWH             24038.461 Hz
FIDRES           0.733596 Hz
AQ              0.6815744 sec
RG                   4096
DW                 20.800 usec
DE                   6.50 usec
TE                  297.0 K
D1             2.00000000 sec
D11            0.03000000 sec
TD0                     1

======== CHANNEL f1 ========
NUC1                  13C
P1                  10.00 usec
PL1                  7.50 dB
SFO1          100.6233325 MHz

======== CHANNEL f2 ========
CPDPRG[2          waltz16
NUC2                   1H
PCPD2               90.00 usec
PL2                  1.40 dB
PL12                17.50 dB
PL13                20.50 dB
SFO2          400.1316005 MHz

F2 - Processing parameters
SI                  32768
SF            100.6127715 MHz
WDW                    EM
SSB      0
LB                   1.00 Hz
GB       0
PC                   1.00
```

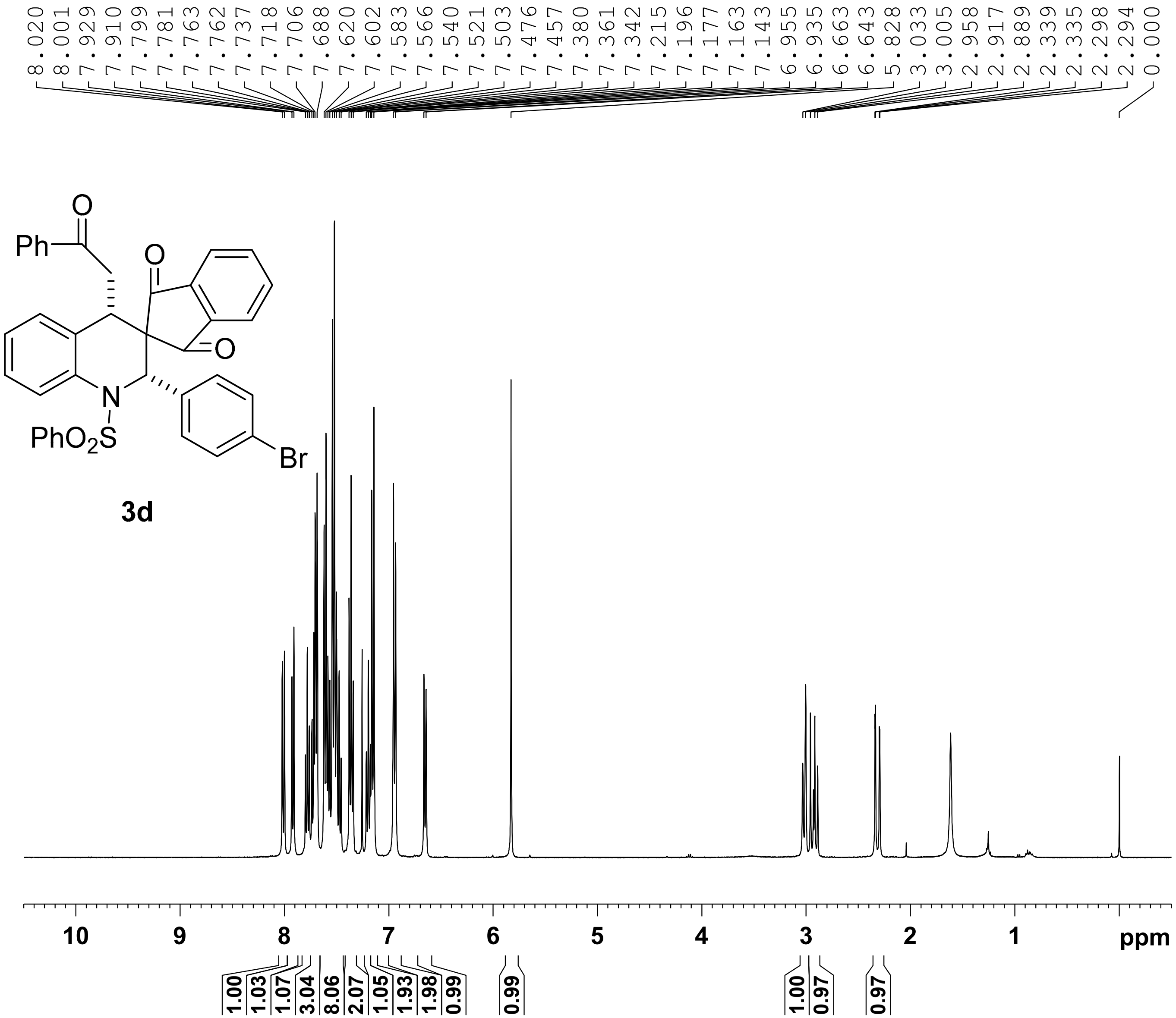


```
Current Data Parameters
NAME                 4-Br
EXPNO                   1
PROCNO                  1

F2 - Acquisition Parameters
Date_            20180804
Time                21.32
INSTRUM             spect
PROBHD   5 mm BBO BB-1H
PULPROG              zg30
TD                  32768
SOLVENT             CDCl3
NS                     16
DS                      0
SWH              7246.377 Hz
FIDRES           0.221142 Hz
AQ              2.2609921 sec
RG                    114
DW                 69.000 usec
DE                   6.50 usec
TE                  296.4 K
D1             2.00000000 sec
TD0                     1

======== CHANNEL f1 ========
NUC1                   1H
P1                  15.00 usec
PL1                  2.20 dB
SFO1          400.1324008 MHz

F2 - Processing parameters
SI                  16384
SF            400.1300110 MHz
WDW                    EM
SSB      0
LB       0 Hz
GB       0
PC                   1.00
```

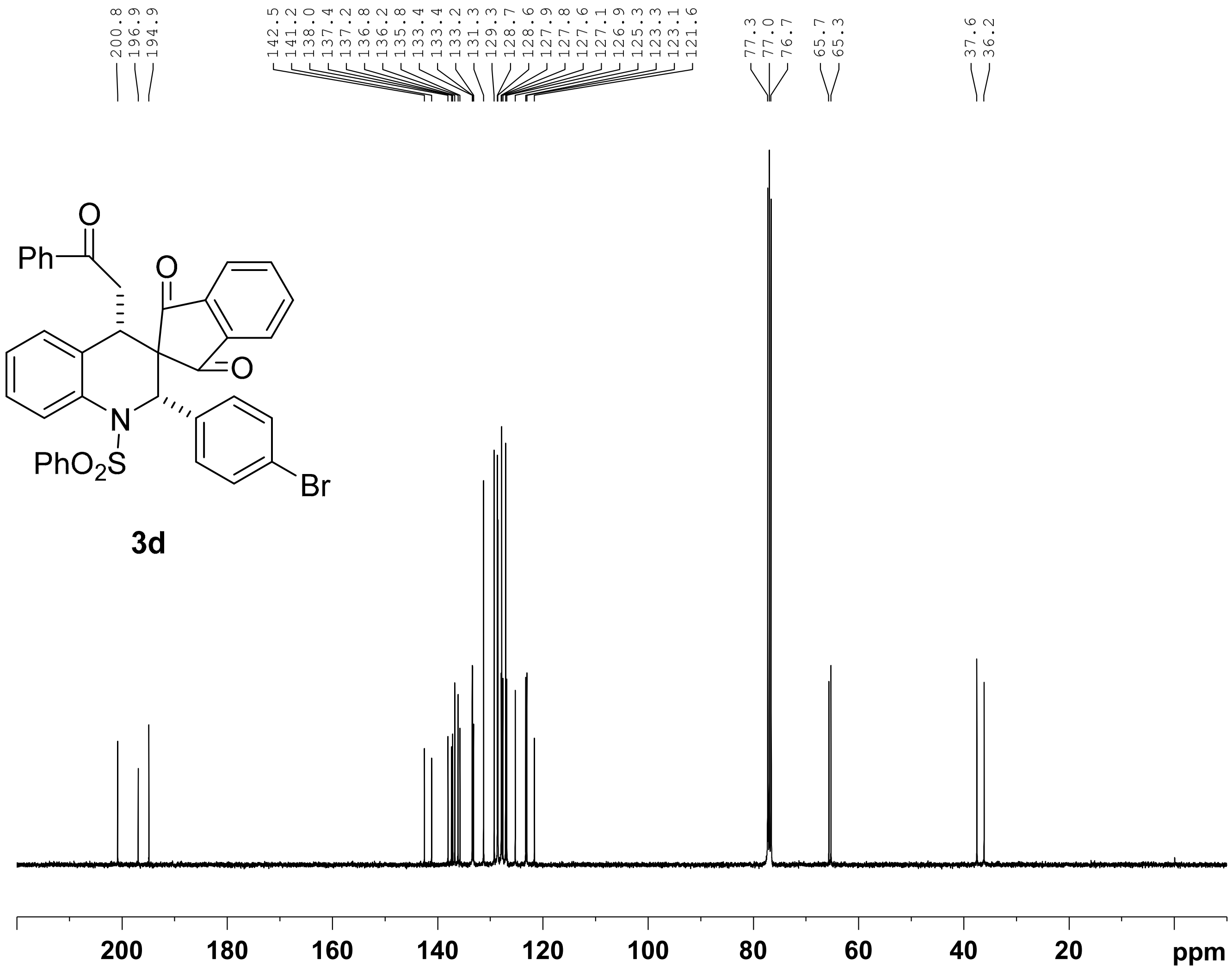


```
Current Data Parameters
NAME                4-Br
EXPNO                 13
PROCNO                 1

F2 - Acquisition Parameters
Date_           20180804
Time               21.35
INSTRUM            spect
PROBHD   5 mm BBO BB-1H
PULPROG          zgpg30
TD                 32768
SOLVENT            CDCl3
NS                  5000
DS                     0
SWH            24038.461 Hz
FIDRES          0.733596 Hz
AQ             0.6815744 sec
RG                  1024
DW                20.800 usec
DE                  6.50 usec
TE                 296.5 K
D1            2.00000000 sec
D11           0.03000000 sec
TD0                    1

======== CHANNEL f1 ========
NUC1                 13C
P1                 10.00 usec
PL1                 7.50 dB
SFO1         100.6233325 MHz

======== CHANNEL f2 ========
CPDPRG[2         waltz16
NUC2                  1H
PCPD2              90.00 usec
PL2                 1.40 dB
PL12               17.50 dB
PL13               20.50 dB
SFO2         400.1316005 MHz

F2 - Processing parameters
SI                 32768
SF           100.6127738 MHz
WDW                   EM
SSB      0
LB                  1.00 Hz
GB       0
PC                  1.00
```

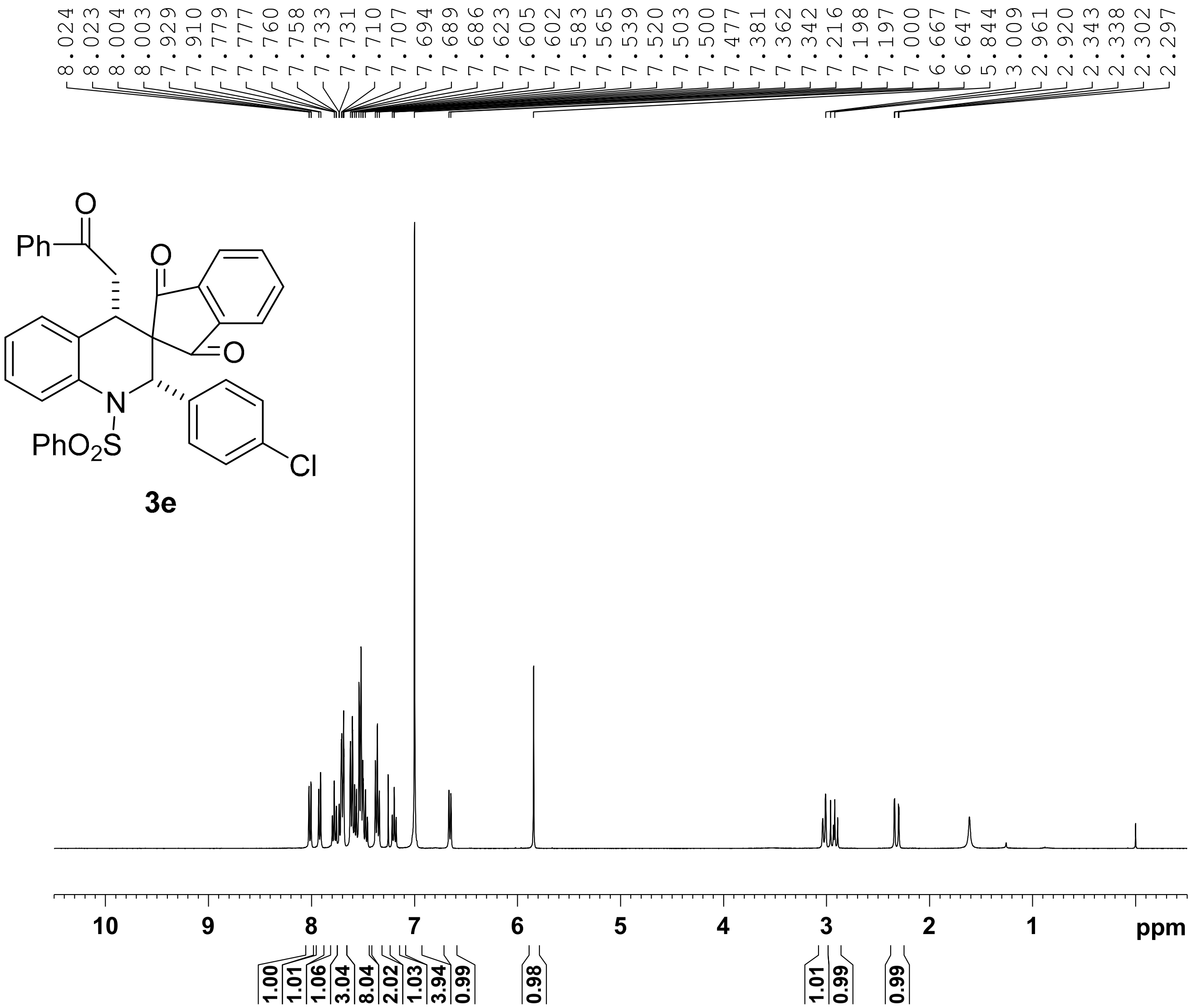


**3e**

| | |
|---|---|
| Current Data Parameters | |
| NAME | 4-Cl |
| EXPNO | 1 |
| PROCNO | 1 |
| F2 - Acquisition Parameters | |
| Date_ | 20180805 |
| Time_ | 14.10 |
| INSTRUM | spect |
| PROBHD | 5 mm BBO BB-1H |
| PULPROG | zg30 |
| TD | 32768 |
| SOLVENT | CDCl3 |
| NS | 16 |
| DS | 0 |
| SWH | 7246.377 Hz |
| FIDRES | 0.221142 Hz |
| AQ | 2.2609921 sec |
| RG | 114 |
| DW | 69.000 usec |
| DE | 6.50 usec |
| TE | 297.3 K |
| D1 | 2.00000000 sec |
| TD0 | 1 |
| ======== CHANNEL f1 ======== | |
| NUC1 | 1H |
| P1 | 15.00 usec |
| PL1 | 2.20 dB |
| SFO1 | 400.1324008 MHz |
| F2 - Processing parameters | |
| SI | 16384 |
| SF | 400.1300111 MHz |
| WDW | EM |
| SSB | 0 |
| LB | 0 Hz |
| GB | 0 |
| PC | 1.00 |

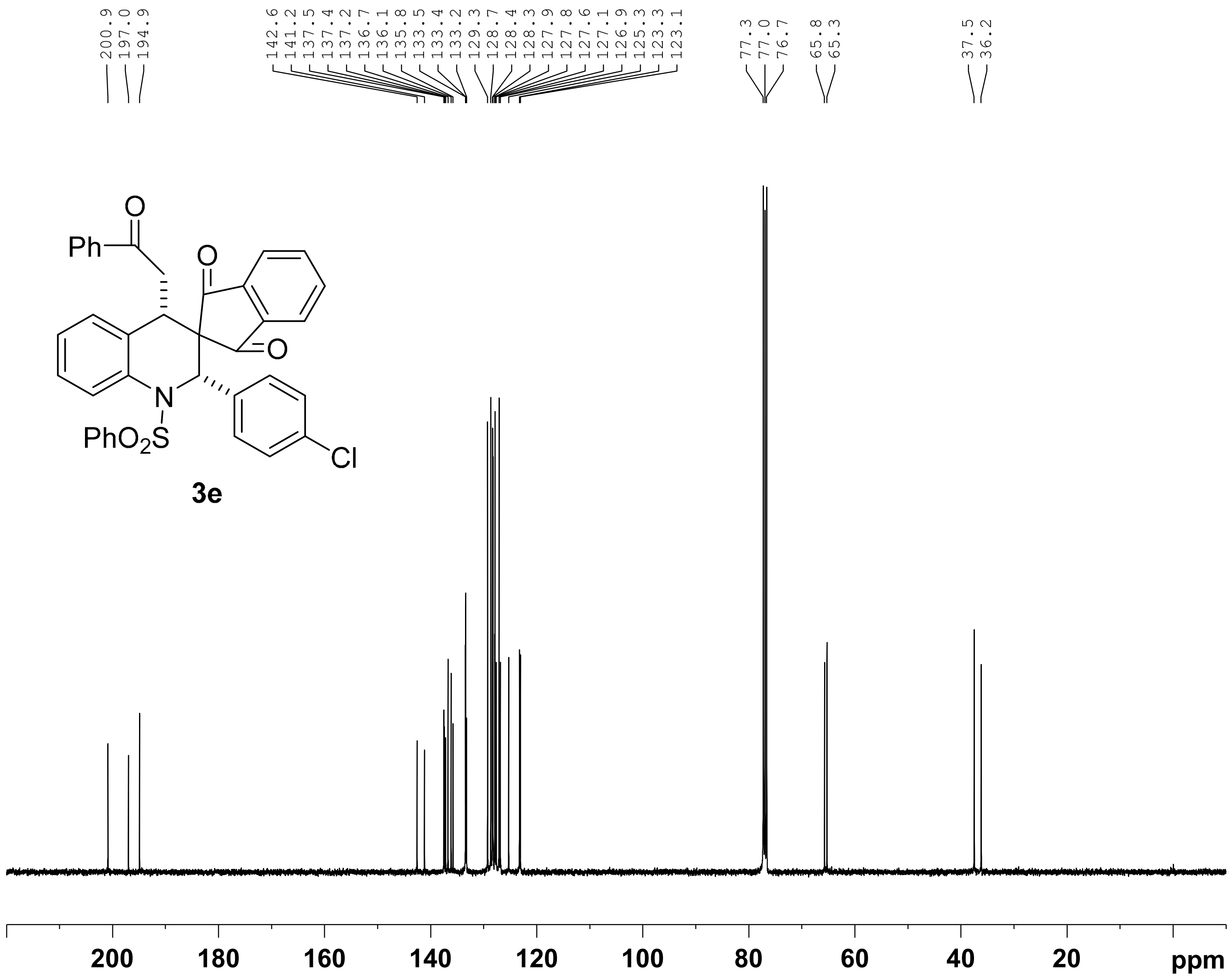

3e



```
Current Data Parameters
NAME                 4-Cl
EXPNO                  13
PROCNO                  1

F2 - Acquisition Parameters
Date_            20180805
Time                14.14
INSTRUM             spect
PROBHD   5 mm BBO BB-1H
PULPROG           zgpg30
TD                  32768
SOLVENT             CDCl3
NS                   5000
DS                      0
SWH            24038.461 Hz
FIDRES          0.733596 Hz
AQ             0.6815744 sec
RG                   2048
DW                 20.800 usec
DE                   6.50 usec
TE                  297.2 K
D1            2.00000000 sec
D11           0.03000000 sec
TD0                     1

======== CHANNEL f1 ========
NUC1                  13C
P1                  10.00 usec
PL1                  7.50 dB
SFO1         100.6233325 MHz

======== CHANNEL f2 ========
CPDPRG[2          waltz16
NUC2                   1H
PCPD2               90.00 usec
PL2                  1.40 dB
PL12                17.50 dB
PL13                20.50 dB
SFO2         400.1316005 MHz

F2 - Processing parameters
SI                  32768
SF           100.6127734 MHz
WDW                    EM
SSB      0
LB                   1.00 Hz
GB       0
PC                   1.00
```

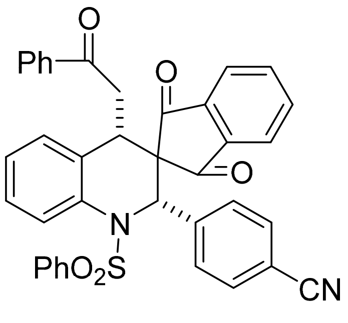

**3f**

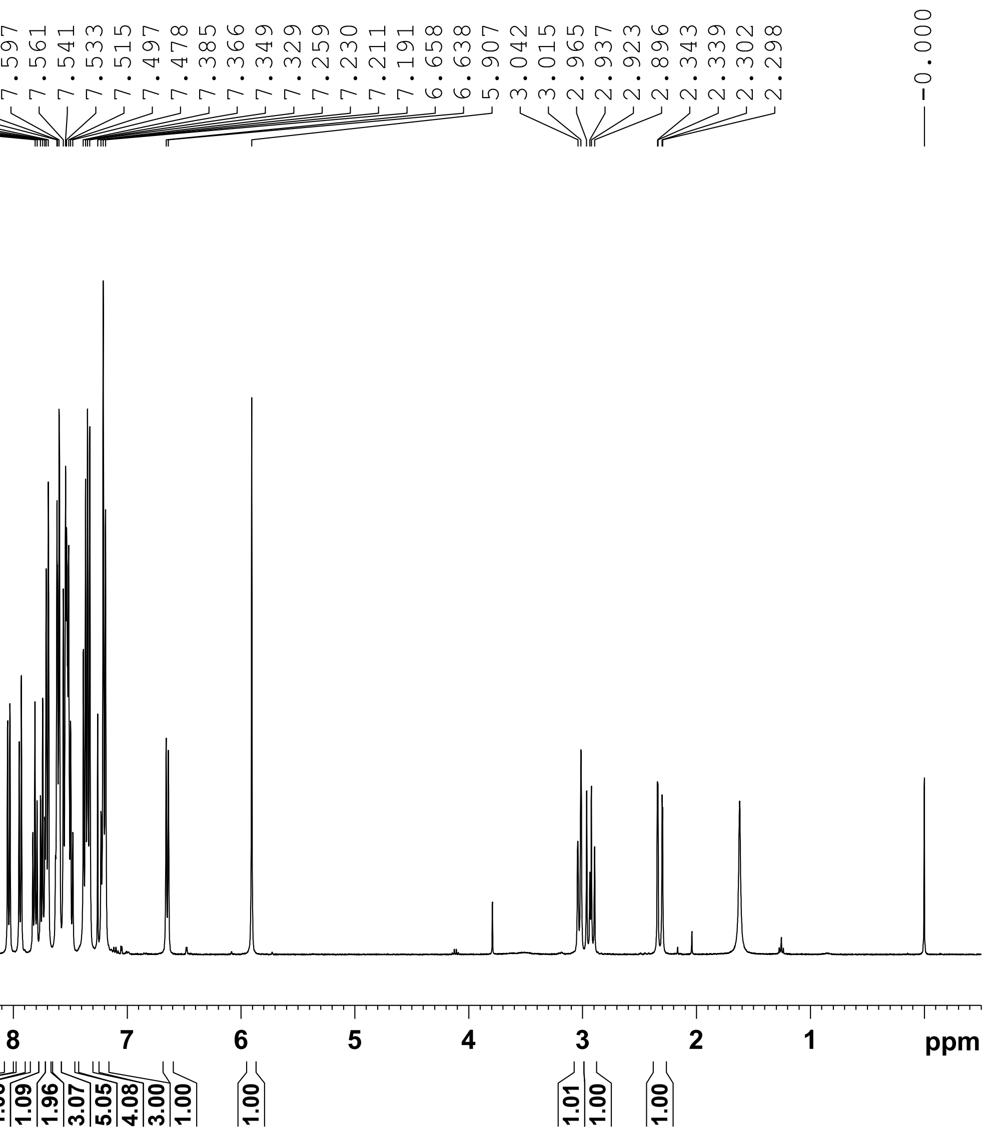


```
Current Data Parameters
NAME              4-CN
EXPNO                1
PROCNO               1

F2 - Acquisition Parameters
Date_          20180803
Time              23.32
INSTRUM           spect
PROBHD    5 mm BBO BB-1H
PULPROG            zg30
TD                32768
SOLVENT           CDCl3
NS                   16
DS                    0
SWH            7246.377 Hz
FIDRES         0.221142 Hz
AQ            2.2609921 sec
RG                  114
DW               69.000 usec
DE                 6.50 usec
TE                296.5 K
D1           2.00000000 sec
TD0                   1

======== CHANNEL f1 ========
NUC1                 1H
P1                15.00 usec
PL1                2.20 dB
SFO1        400.1324008 MHz

F2 - Processing parameters
SI                16384
SF          400.1300098 MHz
WDW                  EM
SSB       0
LB        0 Hz
GB        0
PC                 1.00
```

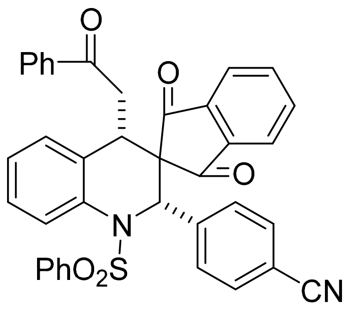

**3f**

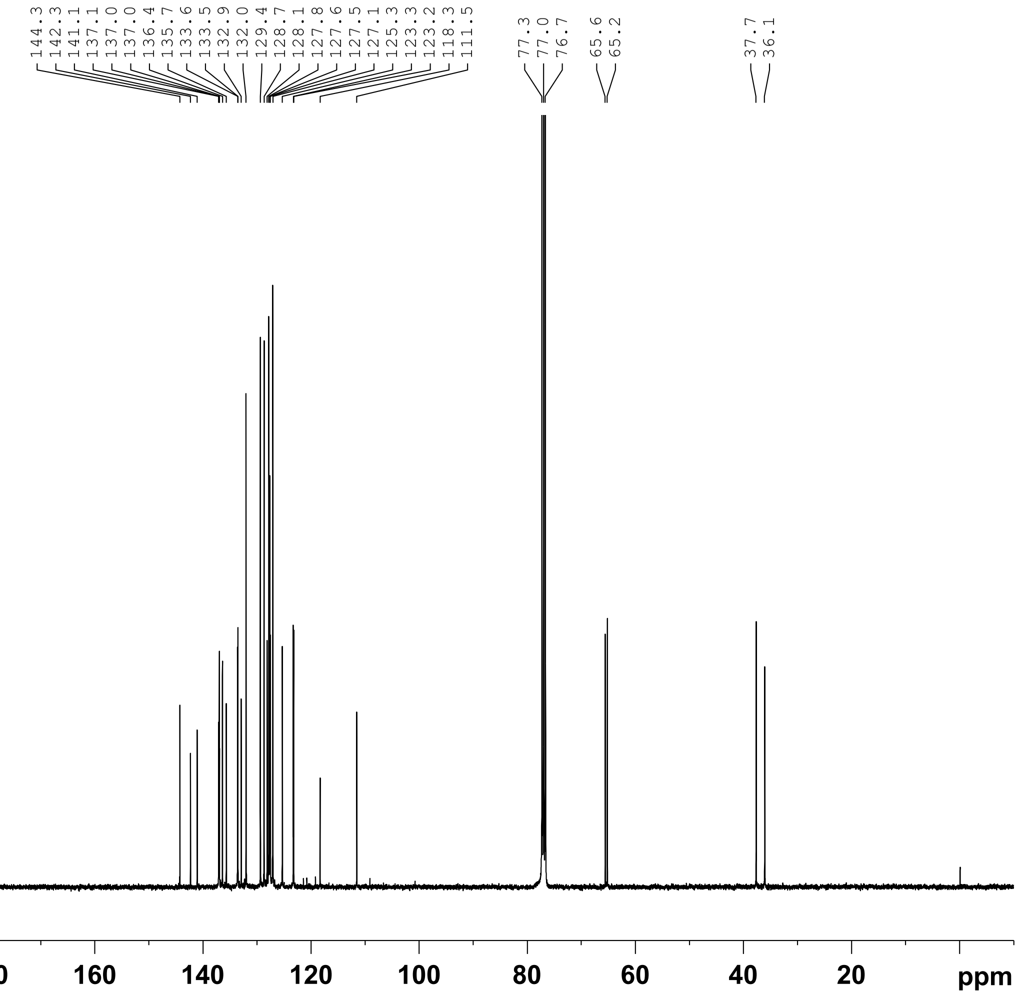


```
Current Data Parameters
NAME                 4-CN
EXPNO                  13
PROCNO                  1

F2 - Acquisition Parameters
Date_            20180803
Time                23.35
INSTRUM             spect
PROBHD   5 mm BBO BB-1H
PULPROG           zgpg30
TD                 32768
SOLVENT            CDCl3
NS                 12781
DS                     0
SWH            24038.461 Hz
FIDRES          0.733596 Hz
AQ             0.6815744 sec
RG                  1024
DW                20.800 usec
DE                  6.50 usec
TE                 296.4 K
D1            2.00000000 sec
D11           0.03000000 sec
TD0                    1

======== CHANNEL f1 ========
NUC1                 13C
P1                 10.00 usec
PL1                 7.50 dB
SFO1         100.6233325 MHz

======== CHANNEL f2 ========
CPDPRG[2         waltz16
NUC2                  1H
PCPD2              90.00 usec
PL2                 1.40 dB
PL12               17.50 dB
PL13               20.50 dB
SFO2         400.1316005 MHz

F2 - Processing parameters
SI                 32768
SF           100.6127738 MHz
WDW                   EM
SSB      0
LB                  1.00 Hz
GB       0
PC                  1.00
```

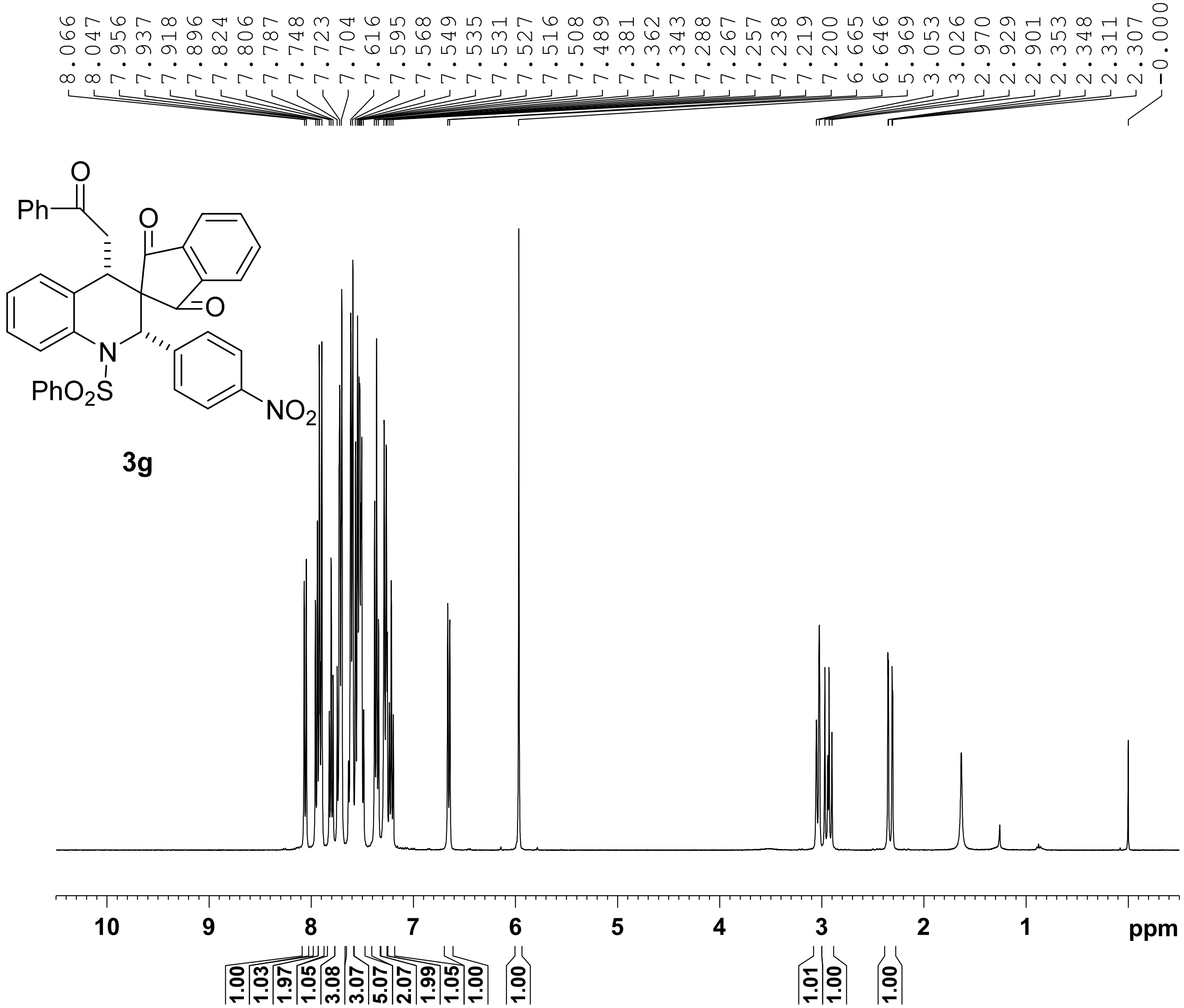


| | |
|---|---|
| Current Data Parameters | |
| NAME | 4-NO2 |
| EXPNO | 1 |
| PROCNO | 1 |
| F2 - Acquisition Parameters | |
| Date_ | 20180730 |
| Time | 9.01 |
| INSTRUM | spect |
| PROBHD | 5 mm BBO BB-1H |
| PULPROG | zg30 |
| TD | 32768 |
| SOLVENT | CDCl3 |
| NS | 16 |
| DS | 0 |
| SWH | 7246.377 Hz |
| FIDRES | 0.221142 Hz |
| AQ | 2.2609921 sec |
| RG | 71.8 |
| DW | 69.000 usec |
| DE | 6.50 usec |
| TE | 296.5 K |
| D1 | 2.00000000 sec |
| TD0 | 1 |
| ======== CHANNEL f1 ======== | |
| NUC1 | 1H |
| P1 | 15.00 usec |
| PL1 | 2.20 dB |
| SFO1 | 400.1324008 MHz |
| F2 - Processing parameters | |
| SI | 16384 |
| SF | 400.1300105 MHz |
| WDW | EM |
| SSB | 0 |
| LB | 0 Hz |
| GB | 0 |
| PC | 1.00 |

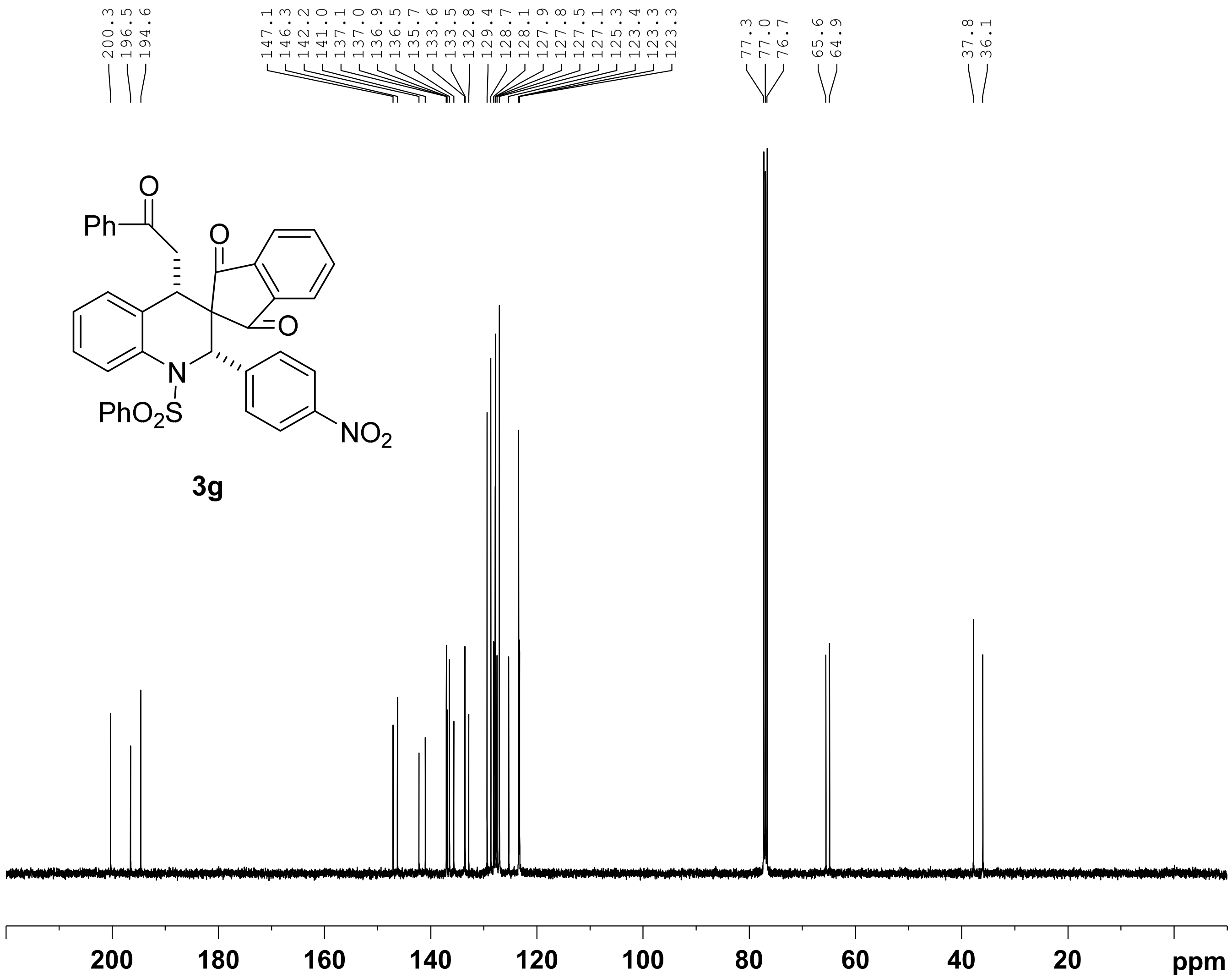


```
Current Data Parameters
NAME               4-NO2
EXPNO                 13
PROCNO                 1

F2 - Acquisition Parameters
Date_           20180730
Time                9.03
INSTRUM            spect
PROBHD   5 mm BBO BB-1H
PULPROG          zgpg30
TD                 32768
SOLVENT            CDCl3
NS                  1129
DS                     0
SWH            24038.461 Hz
FIDRES          0.733596 Hz
AQ             0.6815744 sec
RG                  2048
DW                20.800 usec
DE                  6.50 usec
TE                 296.5 K
D1            2.00000000 sec
D11           0.03000000 sec
TD0                    1

======== CHANNEL f1 ========
NUC1                 13C
P1                 10.00 usec
PL1                 7.50 dB
SFO1         100.6233325 MHz

======== CHANNEL f2 ========
CPDPRG[2         waltz16
NUC2                  1H
PCPD2              90.00 usec
PL2                 1.40 dB
PL12               17.50 dB
PL13               20.50 dB
SFO2         400.1316005 MHz

F2 - Processing parameters
SI                 32768
SF           100.6127764 MHz
WDW                   EM
SSB      0
LB                  1.00 Hz
GB       0
PC                  1.00
```

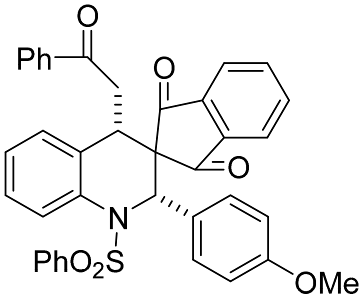


3h

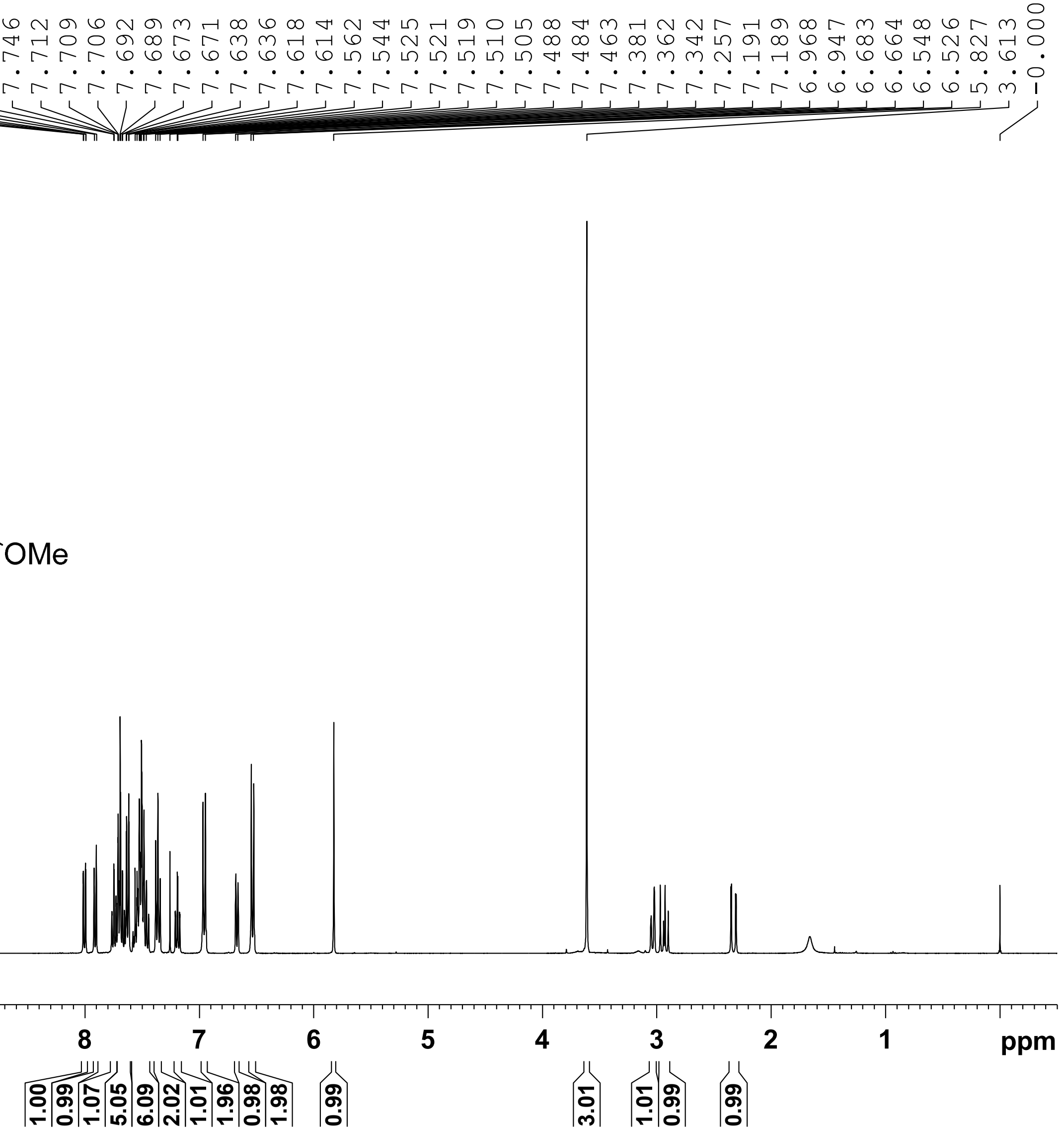


```
Current Data Parameters
NAME              4-OMe
EXPNO                 1
PROCNO                1

F2 - Acquisition Parameters
Date_          20180729
Time              22.29
INSTRUM           spect
PROBHD   5 mm BBO BB-1H
PULPROG            zg30
TD                32768
SOLVENT           CDCl3
NS                   16
DS                    0
SWH            7246.377 Hz
FIDRES         0.221142 Hz
AQ            2.2609921 sec
RG                  114
DW               69.000 usec
DE                 6.50 usec
TE                297.2 K
D1           2.00000000 sec
TD0                   1

======== CHANNEL f1 ========
NUC1                 1H
P1                15.00 usec
PL1                2.20 dB
SFO1        400.1324008 MHz

F2 - Processing parameters
SI                16384
SF          400.1300108 MHz
WDW                  EM
SSB      0
LB       0 Hz
GB       0
PC                 1.00
```
